# Peripapillary Retinal Nerve Fiber Layer Assessment of Spectral Domain Optical Coherence Tomography and Scanning Laser Polarimetry to Diagnose Preperimetric Glaucoma

**DOI:** 10.1371/journal.pone.0108992

**Published:** 2014-10-03

**Authors:** Harsha L. Rao, Ravi K. Yadav, Uday K. Addepalli, Shashikant Chaudhary, Sirisha Senthil, Nikhil S. Choudhari, Chandra S. Garudadri

**Affiliations:** VST Glaucoma Center, L V Prasad Eye Institute, Banjara Hills, Hyderabad, India; Bascom Palmer Eye Institute, University of Miami School of Medicine, United States of America

## Abstract

**Purpose:**

To compare the abilities of peripapillary retinal nerve fiber layer (RNFL) parameters of spectral domain optical coherence tomograph (SDOCT) and scanning laser polarimeter (GDx enhanced corneal compensation; ECC) in detecting preperimetric glaucoma.

**Methods:**

In a cross-sectional study, 35 preperimetric glaucoma eyes (32 subjects) and 94 control eyes (74 subjects) underwent digital optic disc photography and RNFL imaging with SDOCT and GDx ECC. Ability of RNFL parameters of SDOCT and GDx ECC to discriminate preperimetric glaucoma eyes from control eyes was compared using area under receiver operating characteristic curves (AUC), sensitivities at fixed specificities and likelihood ratios (LR).

**Results:**

AUC of the global average RNFL thickness of SDOCT (0.786) was significantly greater (p<0.001) than that of GDx ECC (0.627). Sensitivities at 95% specificity of the corresponding parameters were 20% and 8.6% respectively. AUCs of the inferior, superior and temporal quadrant RNFL thickness parameters of SDOCT were also significantly (p<0.05) greater than the respective RNFL parameters of GDx ECC. LRs of outside normal limits category of SDOCT parameters ranged between 3.3 and 4.0 while the same of GDx ECC parameters ranged between 1.2 and 2.1. LRs of within normal limits category of SDOCT parameters ranged between 0.4 and 0.7 while the same of GDx ECC parameters ranged between 0.7 and 1.0.

**Conclusions:**

Abilities of the RNFL parameters of SDOCT and GDx ECC to diagnose preperimetric glaucoma were only moderate. Diagnostic abilities of the RNFL parameters of SDOCT were significantly better than that of GDx ECC in preperimetric glaucoma.

## Introduction

Spectral domain optical coherence tomography (SDOCT) and scanning laser polarimetry (SLP) are the two currently used common imaging techniques for peripapillary retinal nerve fiber layer (RNFL) evaluation in glaucoma. SDOCT is a recent technique which enables imaging the ocular structures with higher resolution and faster scan rate compared to the previous version of this technology (Stratus OCT, Carl Zeiss Meditec, Inc., Dublin, CA) [1], [2]. GDx (Carl Zeiss Meditec Inc. Dublin, CA), the commonly used SLP device measures the RNFL birefringence in vivo and is based on the principle that polarized light passing through the birefringent RNFL undergoes a measurable phase shift, known as retardation, which is linearly related to the RNFL tissue thickness [3]. The current SLP protocol, called the enhanced corneal compensation (ECC), optimizes imaging by improving the signal-to-noise ratio compared to the previous version (GDx Variable Corneal Compensation) [4]–[6].

Though numerous studies have reported good diagnostic ability of both SDOCT [7]–[11] and GDx ECC [12]–[14] in glaucoma, there is limited literature on head to head comparison of these imaging techniques in the same population [15], [16]. Also, most of these studies have employed a case-control design including glaucoma patients (cases), defined based on the presence of repeatable characteristic glaucomatous visual field (VF) defects; and normal subjects (controls), usually recruited from the general population and having normal intraocular pressures (IOP), healthy appearance of the optic nerve and normal VFs. However, in clinical practice, a diagnostic test is used to rule-in disease in very early stages (preferably in preperimetric stages of glaucoma) or rule-out disease in subjects suspected of having disease. In a previous study, we compared the diagnostic abilities of the RNFL parameters of SDOCT and GDx ECC in perimetric glaucoma and found them to be comparable [17]. However there were indications of SDOCT being better in early stages of perimetric glaucoma. With this background, the purpose of the current study was to compare the abilities of RNFL parameters of SDOCT and GDx ECC in detecting preperimetric glaucoma.

## Methods

This was an observational, cross-sectional study of consecutive subjects referred by general ophthalmologists to a tertiary eye care facility between September 2010 and November 2012 as glaucoma suspects based on the optic disc appearance. Written informed consent was obtained from all participants and the Ethics Committee of L V Prasad Eye Institute approved the methodology. All methods adhered to the tenets of the Declaration of Helsinki for research involving human subjects.

Inclusion criteria were age ≥18 years, best corrected visual acuity of 20/40 or better and refractive error within ±5.0 D sphere and ±3 D cylinder. Exclusion criteria were presence of any media opacities that prevented good imaging and any retinal (including macular) or neurological diseases other than glaucoma which could confound the results of visual field examination and RNFL measurements with SDOCT or SLP. All participants underwent a comprehensive ocular examination which included a detailed medical history, best corrected visual acuity measurement, slit-lamp biomicroscopy, Goldmann applanation tonometry, gonioscopy, dilated fundus examination, digital optic disc photography, standard automated perimetry (SAP) and RNFL imaging with SDOCT and SLP.

SAP was performed using a Humphrey Field analyzer, model 750 (Zeiss Humphrey Systems, Dublin, CA), with the Swedish interactive threshold algorithm (SITA) standard 24-2 program. VFs with fixation losses, false positive and false negative response rates of less than 20% were considered reliable. VFs were considered glaucomatous if the pattern standard deviation had a P value of less than 5% and the glaucoma hemifield test result was outside normal limits [18]. VFs were considered normal if the above 2 criteria were not satisfied. For the study, we included all subjects with reliable and normal SAP results.

Digital optic disc photographs were obtained by trained technicians (FF 450plus, Carl Zeiss Meditec Systems GmbH, Pirmasens, Germany). Photographs consisted of a 50 degree image centered on the optic disc, a similar image centered on the macula, a 30 degree image centered on the optic disc and a 20 degree image centered on the disc. All these images also consisted of one colored and one red-free image each. The photographs were evaluated by two experts independently, who were masked to the clinical examination results of the subjects and also the results of visual field and imaging examinations. Experts classified the optic disc photographs into glaucomatous and non-glaucomatous based on the presence of focal or diffuse neuroretinal rim thinning, localized notching or nerve fiber layer defects. Discrepancies between the two experts were resolved by consensus. Eyes, where a classification to either glaucoma or control group was not possible by both the experts, were labeled as “suspects” and excluded from the analysis.

SDOCT examination was performed with the RTVue (software version 5.1.0.90). RTVue uses a scanning laser diode with a wavelength of 840±10 nm to provide images of ocular microstructures. The protocol used for RNFL imaging with RTVue in this study was ONH (optic nerve head) scan. This protocol has been explained earlier [19], [20]. In brief, ONH protocol consists of 12 radial scans 3.4 mm in length and 6 concentric ring scans ranging from 2.5 to 4.0 mm in diameter all centered on the optic disc. ONH protocol calculates various parameters that describe the ONH and also generates a polar RNFL thickness map which is the RNFL thickness measured along a circle of 3.45 mm in diameter centered on the optic disc. The RNFL parameters generated and used in the study were temporal average (temporal 90 degree), superior average (superior 90 degree), nasal average (nasal 90 degree), inferior average (inferior 90 degree) and global average (over 360 degree). The RNFL parameters are also compared with the internal normative database of 861 eyes of different ethnicities within the software and one of the three diagnostic categorizations is provided. “Outside normal limits” categorization indicates that the value is lesser than the lower 99% confidence interval (CI) of the healthy, age-matched population. “Borderline” result indicates that the value is between the 95% and 99% CI, and a “within-normal-limits” indicates that the value is within the 95% CI. Only well-centered images with a signal strength index (SSI) of ≥30 were used for analysis. RNFL segmentation was inspected manually by one of the experts (HLR) and eyes in which the segmentation algorithm failed were excluded.

SLP examination was done using GDxPRO (version 1.1.1, Carl Zeiss Meditec Inc. Dublin, CA). The general principles of SLP and the algorithm used for ECC have been described in detail previously [3], [5], [12]. A corneal scan was obtained in all the participants before the RNFL scan to measure the corneal birefringence. Scan image was checked for focus, alignment and movement artifacts. All corneal scans had a quality score of ≥8. Following corneal scan acquisition, the macular ellipse was manually centered on the macular bow tie pattern. Macular bow tie pattern was well defined in all the eyes. RNFL was imaged using a single scan protocol. A fixed concentric measurement band centered on the optic disc with a 3.2-mm outer and a 2.4-mm inner diameter was used to generate the peripapillary retardation measurements. The parameters generated on the printout and used in this study were temporal average (temporal 50 degree), superior average (superior 120 degree), nasal average (nasal 70 degree), inferior average (inferior 120 degree), TSNIT (temporal superior nasal inferior and temporal) average (over 360 degree) and nerve fiber indicator (NFI). The NFI is a support vector machine score based on several RNFL measures and assigns a number from 0 to 100 to each eye. The higher the NFI, the greater is the likelihood of having glaucoma. NFI value of less than 30 is considered “within normal limits”, 30 to 50 is considered “borderline” and more than 50 is considered “outside normal limits”. The superior, inferior and TSNIT average are also compared with the internal normative database of 251 subjects within the software and one of the three diagnostic categorizations as with SDOCT is provided. Only well-focused, centered and illuminated images with a quality score of ≥7, typical scan score (TSS)>80 and a residual anterior segment retardation of ≤4 were included for analysis.

Participants had all examinations as well as RNFL imaging with SDOCT and SLP performed on the same day.

### Statistical analysis

Descriptive statistics included mean and standard deviation for normally distributed variables and median and inter-quartile range (IQR) for non-normally distributed variables. Receiver operating characteristic (ROC) curves were used to describe the ability of RNFL parameters of both SDOCT and GDx ECC to discriminate glaucomatous eyes from control eyes. Sensitivities at fixed specificities of 80% and 95% were determined for all the parameters. To obtain confidence intervals for area under the ROC curves (AUC) and sensitivities, a bootstrap re-sampling procedure was used (n = 1000 re-samples). As measurements from both eyes of the same subject are likely to be correlated, the standard statistical methods for parameter estimation lead to underestimation of standard errors and to confidence intervals that are too narrow [21]. Therefore, the cluster of data for the study subject was considered as the unit of resampling and bias corrected standard error was calculated during all estimations. This procedure has been used in literature to adjust for the presence of multiple correlated measurements from the same unit [22], [23]. Likelihood ratios (LR) were reported for diagnostic categorization (outside normal limits, borderline, or within normal limits) provided after comparison with the instrument's internal normative database. LR is the probability of a given test result in those with disease divided by the probability of the same test result in those without the disease [24]–[26]. The LR for a given test result indicates how much that result will raise or lower the probability of disease. A LR of 1 or close to 1 would mean that the test provides no additional information about the post-test probability of the disease. LRs higher than 10 or lower than 0.1 would be associated with large effects on post-test probability, LRs from 5 to 10 or from 0.1 to 0.2 would be associated with moderate effects, LRs from 2 to 5 or from 0.2 to 0.5 would be associated with small effects [24]. The 95% CIs for LRs were calculated according to the method proposed by Simel et al. [27].

Statistical analyses were performed using commercial software (Stata ver. 12.1; StataCorp, College Station, TX). A p value of <0.05 was considered statically significant.

## Results

**Figure 1:**
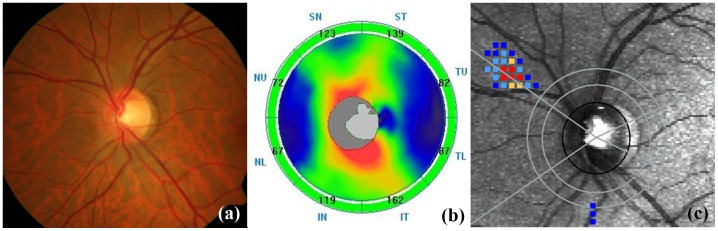
Representative image of an eye in the control group showing normal optic disc and retinal nerve fiber layer (RNFL) on disc photograph (a), normal RNFL thickness on spectral domain optic coherence tomography (b) and normal RNFL birefringence on GDx enhanced corneal compensation (c).

**Figure 2:**
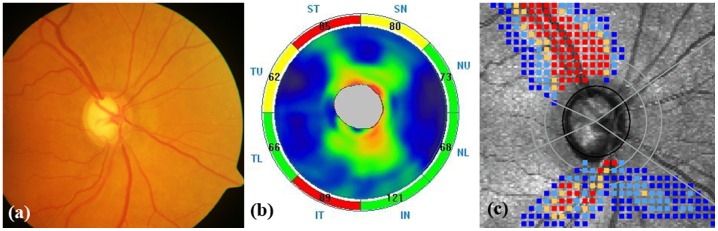
Representative image of an eye in the preperimetric glaucoma group showing glaucomatous optic disc with retinal nerve fiber layer (RNFL) defects superiorly and inferiorly (a), corresponding RNFL thickness abnormalities on spectral domain optic coherence tomography (b) and corresponding abnormal RNFL birefringence on GDx enhanced corneal compensation (c).

Three hundred and forty three eyes of 255 consecutive subjects referred for glaucoma evaluation to our center were analyzed. Twenty eight eyes with unreliable VFs, 2 eyes with poor quality disc photographs, 9 eyes with SSI of less than 30 on SDOCT and 28 eyes with either a quality score of <7, TSS value of <80 or residual anterior segment retardation of>4 on GDx ECC were excluded. Of the remaining, 121 eyes with glaucomatous VFs were excluded, leaving behind 155 eyes with normal VFs. Of these, 26 eyes classified as “disc suspects” by both glaucoma experts were excluded. Of the remaining 129 eyes, 35 eyes of 32 subjects with the optic disc classification as “glaucoma” formed the preperimetric glaucoma group and 94 eyes of 74 subjects with optic disc classification as “non-glaucoma” formed the control group. Table 1 shows that age and visual field parameters of the two groups were comparable. Glaucoma patients had significantly smaller optic discs than the control subjects. Figure 1 shows normal optic disc and RNFL on disc photograph (a), normal RNFL thickness on SDOCT (b) and normal RNFL birefringence on GDx ECC (c) in an eye in the control group. Figure 2 shows an eye in the preperimetric glaucoma group with glaucomatous optic disc and RNFL defects superiorly and inferiorly on disc photograph (a), corresponding RNFL thinning on SDOCT (b) and corresponding RNFL birefringence abnormality on GDx ECC (c). VFs were normal in both the above eyes.

**Table 1:** Demographic and visual field characteristics of the patients.

	Control group (n = 94 eyes)	Glaucoma group (n = 35 eyes)	P value
**Age (years)**	51±13	53±12	0.61
**Disc area (mm^2^)**	2.21 (1.84, 2.55)	1.97 (1.45, 2.32)	0.02
**Mean deviation (dB)**	−1.68 (−2.71, −0.71)	−2.13 (−3.10, −0.84)	0.35
**Pattern standard deviation (dB)**	1.69 (1.53, 2.08)	1.74 (1.54, 2.25)	0.54
**Visual field index (%)**	99 (98, 99)	99 (97, 99)	0.33

dB: decibel. All values are median and inter-quartile ranges unless specified.

Table 2 shows the RNFL parameters of SDOCT in the two groups of participants. All RNFL parameters of SDOCT were significantly thinner in the glaucoma group compared to the control group. AUC and sensitivities at fixed specificities after adjusting for the differences in the disc area between the 2 groups using covariate-adjustment, as proposed by Pepe [28], are also shown in Table 2. Average, inferior and temporal RNFL thickness parameters showed the best AUCs and sensitivities at fixed specificities of 95% and 80%.

**Table 2:** Mean values of SDOCT retinal nerve fiber layer thickness parameters in glaucoma and control eyes with areas under the receiver operating characteristic curves and sensitivities at fixed specificities.

Retinal nerve fiber layer parameters (in microns)	Control (Mean ± SD)	Glaucoma (Mean ± SD)	P value	AUC	Sensitivity at 95% specificity	Sensitivity at 80% specificity
**Signal strength index**	51±10	51±9	0.98			
**Temporal quadrant**	79.0±10.8	69.8±9.7	<0.001	0.762 (0.631–0.851)	28.6% (2.6–53.2)	62.9% (43.8–80.5)
**Superior quadrant**	123.9±19.1	109.2±15.1	<0.001	0.701 (0.585–0.797)	22.9% (8.8–45.8)	45.7% (25.8–67.5)
**Nasal quadrant**	79.6±12.0	72.9±14.6	0.01	0.604 (0.486–0.731)	25.7% (12.8–44.7)	42.9% (26.4–62.1)
**Inferior quadrant**	128.2±20.1	108.9±17.4	<0.001	0.752 (0.634–0.842)	22.9% (7.7–45.7)	57.1% (30.4–80.5)
**Average thickness**	102.7±11.6	90.2±9.7	<0.001	0.786 (0.676–0.866)	20.0% (3.4–40.0)	45.7% (19.4–68.4)

SDOCT: spectral domain optical coherence tomograph; SD: standard deviation; AUC: area under the receiver operating characteristic curve; Figures in brackets are 95% confidence intervals. AUCs and sensitivities are adjusted for the difference in disc area between the control and glaucoma group.

Table 3 shows the RNFL parameters of GDx ECC in the two groups of participants. Quality score and TSS were comparable between the groups, while residual anterior segment retardance was more in the glaucoma group. All RNFL parameters except the temporal and nasal quadrant RNFL measurements were significantly lesser in the glaucoma group. NFI value was significantly higher in the glaucoma group. AUC and sensitivities at fixed specificities after adjusting for the differences in the disc area between the 2 groups are also shown in Table 3. NFI and inferior quadrant RNFL parameter showed the best AUCs.

**Table 3:** Mean values of GDx ECC retinal nerve fiber layer parameters in glaucoma and control eyes with areas under the receiver operating characteristic curves and sensitivities at fixed specificities.

Retinal nerve fiber layer parameters (in microns)	Control (Mean ± SD)	Glaucoma (Mean ± SD)	P value	AUC	Sensitivity at 95% specificity	Sensitivity at 80% specificity
Quality score*	9 (9, 9)	9 (9, 9)	0.77			
Typical scan score*	100 (99, 100)	100 (99, 100)	0.94			
Residual retardation*	1 (0, 2)	1 (1, 3)	0.03			
Temporal quadrant	19.8±6.6	19.9±7.9	0.88	0.521 (0.393–0.645)	11.4% (2.9–30.0)	28.6% (7.1–54.8)
Superior quadrant	56.7±10.2	52.1±10.0	0.02	0.598 (0.493–0.692)	11.4% (2.6–23.1)	22.9% (7.9–38.1)
Nasal quadrant	34.2±7.8	31.4±8.2	0.07	0.622 (0.517–0.743)	8.6% (2.4–19.2)	28.6% (8.0–54.8)
Inferior quadrant	57.7±9.9	52.2±10.4	0.01	0.632 (0.518–0.728)	8.6% (2.3–22.2)	28.6% (11.4–50.0)
Average	47.5±6.6	43.5±7.1	0.004	0.627 (0.513–0.728)	8.6% (2.0–18.5)	37.1% (22.2–54.8)
NFI*	27 (17, 42)	39 (28, 54)	0.001	0.671 (0.580–0.780)	14.3% (2.9–28.6)	34.3% (16.3–55.6)

ECC: enhanced corneal compensation; SD: standard deviation; AUC: area under the receiver operating characteristic curve; CI: confidence interval; NFI: nerve fiber indicator; *median with inter-quartile range. AUCs and sensitivities are adjusted for the difference in disc area between the control and glaucoma group.

**Figure 3:**
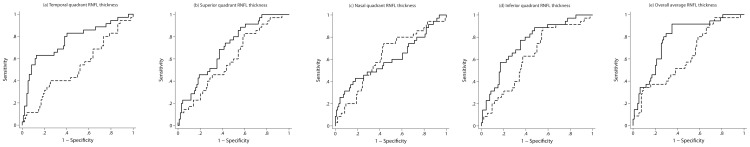
Receiver operating characteristic curves of the (a) temporal quadrant, (b) superior quadrant, (c) nasal quadrant, (d) inferior quadrant and (e) overall average retinal nerve fiber layer (RNFL) thickness parameters of spectral domain optical coherence tomography (solid line) and GDx enhanced corneal compensation (dashed line) in preperimetric glaucoma.

Except for the nasal quadrant measurements (p = 0.77), AUCs of all SDOCT RNFL measurements were significantly greater (p<0.05) than the corresponding GDx ECC measurements in diagnosing preperimetric glaucoma. Figure 3 shows the ROC curves of the quadrant and overall average RNFL thickness parameters of SDOCT and GDx ECC.

Table 4 shows the LRs associated with the diagnostic categorization of the SDOCT and GDx ECC parameters after comparison with the respective instrument's internal normative database. Outside normal limits categories of SDOCT parameters were associated with small effects on the post-test probability of disease while that of GDx ECC parameters were associated with no effects. Within normal limits category of inferior quadrant RNFL average of SDOCT was associated with small effects on the post-test probability of disease. Within normal limits category of other SDOCT parameters and all GDx ECC parameters, and borderline categories of the RNFL parameters of both SDOCT and GDx ECC were associated with no effect on the post-test probability of glaucoma.

**Table 4:** Likelihood ratios (with 95% confidence interval)* of the normative database classification of SDOCT and GDx ECC parameters to discriminate glaucoma from control eyes.

RNFL Parameter	Within normal limits		Borderline		Outside normal limits	
	SDOCT	GDx ECC	SDOCT	GDx ECC	SDOCT	GDx ECC
Superior quadrant	0.68 (0.49–0.94)	0.95 (0.75–1.21)	1.05 (0.51–2.16)	1.04 (0.48–2.27)	3.26 (1.52–6.98)	1.19 (0.55–2.58)
Inferior quadrant	0.45 (0.27–0.74)	0.67 (0.45–1.00)	1.40 (0.65–3.01)	1.12 (0.60–2.10)	3.73 (2.10–6.62)	2.10 (1.16–3.80)
Average	0.66 (0.46–0.94)	0.71 (0.49–1.03)	2.44 (1.28–4.66)	0.81 (0.35–1.85)	3.99 (1.75–9.12)	1.79 (1.05–3.04)
Nerve fiber indicator		0.73 (0.52–1.04)		1.01 (0.52–1.96)		1.97 (1.05–3.72)

RNFL: retinal nerve fiber layer; SDOCT: spectral domain optical coherence tomograph; ECC: enhanced corneal compensation; SD: standard deviation.

* analysis based on number of eyes.

## Discussion

In this study to compare the abilities of RNFL parameters of SDOCT and GDx ECC in diagnosing preperimetric glaucoma, we found that most of the SDOCT RNFL parameters were significantly better than the corresponding GDx ECC parameters.

Recent studies have compared the RNFL parameters of SDOCT and GDx ECC in perimetric glaucoma and have found that the diagnostic abilities were comparable [15]–[17]. Garas et al.[16] though had a group of preperimetric glaucoma subjects in their study, did not analyze the results separately in this subgroup.

It is important to note that the RNFL quadrants on the printouts of SDOCT and GDx ECC are not exactly similar. The 4 RNFL quadrants of SDOCT are 90 degrees each while the superior and inferior quadrants of GDx ECC are 120 degrees, temporal quadrant is 50 degrees and nasal quadrant is 70 degrees. This might have resulted in some bias during comparison. But considering the fact that the parameters are used in clinical practice as in the printout, we decided to analyze them as such.

There are no studies in literature evaluating the diagnostic ability of GDx ECC in preperimetric glaucoma. There are however, studies evaluating the diagnostic ability of RNFL parameters of SDOCT in preperimetric glaucoma and the AUCs and sensitivities reported in these studies are better than those found in our study [29]–[35]. This is probably because of the difference in the control group of our study compared to other studies. The control group in our study was selected from the group of subjects referred as glaucoma suspects based on their optic disc appearance by general ophthalmologists. These subjects were however diagnosed as normals based on the optic disc evaluation by glaucoma experts and the normal VFs. Therefore in true sense, optic discs included in the control group though were referred as suspects for glaucoma, were not true suspects but were the ones that caused a diagnostic uncertainty among general ophthalmologists. Significantly larger disc size in the control group suggests that these were the eyes with large discs and large physiologic cups, mislabeled as “glaucoma suspects” by general ophthalmologists. There was however no ambiguity in their classification by the glaucoma experts. In case of ambiguity, optic discs were called “suspects” and excluded from the analysis. We believe that including a control group which is likely to cause some amount of diagnostic uncertainty is more meaningful and mimics the real-life clinical situation than a control group with no suspicious findings of the disease as was used in the previous studies. We have earlier reported the effect of such a control group on the diagnostic ability of SDOCT in early glaucoma [20] and preperimetric glaucoma [36]. There was also no ambiguity in the classification of preperimetric glaucoma eyes as two glaucoma experts agreed upon the presence of glaucomatous structural changes. Also upon unmasking the eyes after analysis, of the 29 subjects who had contributed one eye to the preperimetric glaucoma group, 23 (79%) had perimetric glaucoma in the fellow eye.

We found that the AUC and sensitivity at fixed specificities of temporal quadrant RNFL thickness with SDOCT was comparable to that of the inferior quadrant and average RNFL thickness. All previous studies with SDOCT have reported the diagnostic ability of temporal RNFL thickness to be significantly lower than that of the inferior quadrant and average RNFL thickness [7]–[11]. Though the reason for this finding in our study is unclear, it may be related to the local change in the RNFL that happens in the papillomacular bundle in preperimetric glaucoma. Future studies correlating the temporal RNFL thickness change with the macular RNFL thickness change may be able to conclusively assess if this result is a true or a chance finding.

In addition to sensitivity, specificity and AUC, diagnostic tests can also be summarized in terms of LR. LR is higher than the other measures in hierarchy, as it expresses the magnitude by which the probability of a diagnosis in a given patient is modified by the results of the test [37]–[39]. In other words, the LR indicates how much a given diagnostic test result will change (increase or decrease) the pretest probability of the disease. We therefore evaluated the LRs associated with the diagnostic categorization of the RNFL parameter value, after comparison with the instrument's normative database. The magnitudes of the LRs associated with the outside normal limits category of SDOCT RNFL parameters were associated with small effects on the post-test probability of disease while that of GDx ECC parameters were associated with no effects. The “outside normal limits” category of SDOCT appeared to be better in “ruling in” glaucoma compared to that of GDX ECC. Within normal limits category of the inferior quadrant RNFL parameter of SDOCT was associated with small effects on the post-test probability of disease while that of all GDx ECC parameters were associated with no effects. This would mean that the “within normal limits” category of SDOCT may be better than GDx ECC in “ruling out” glaucoma. “Borderline” categories of both the devices were associated with no effects on the post-test probability of glaucoma, meaning that they were not useful either in “ruling in” or “ruling out” glaucoma. It should, however be noted that, in clinical practice even small effects on post-test probability may be relevant and useful, depending on the overall clinical picture and the pretest probability of disease.

A limitation of our study which might have inflated the diagnostic accuracy results, is the use of structural change on disc photographs as the gold standard while testing the technologies that once again evaluate the structural changes in glaucoma [40]. However, this is related to the lack of a good non-structural, reference standard for diagnosing glaucoma at this point in time in patients with normal SAP results. Though this might have affected the diagnostic accuracies, the effect on the comparison between devices may be minimal.

In conclusion, the abilities of RNFL parameters of SDOCT and GDx ECC to diagnose preperimetric glaucoma were only moderate. Diagnostic abilities of the RNFL parameters of SDOCT were significantly better than that of GDx ECC in preperimetric glaucoma.
